# Basal Anti Mullerian hormone levels and endometrial thickness at midcycle can predict the outcome after clomiphene citrate stimulation in anovulatory women with PCOS, a retrospective study

**DOI:** 10.1007/s00404-019-05359-7

**Published:** 2019-11-06

**Authors:** Aulona Gaba, Steffen Hörath, Marlene Hager, Rodrig Marculescu, Johannes Ott

**Affiliations:** 1 Department of Obstetrics and Gynecology, Clinical Division of Obstetrics and Maternal-Fetal Medicine, Medical University of Vienna, Vienna, Austria; 2 Department of Obstetrics and Gynecology, Clinical Division of Gynecologic Endocrinology and Reproductive Medicine, Medical University of Vienna, Spitalgasse 23, 1090 Vienna, Austria; 3 Department of Laboratory Medicine, Medical University of Vienna, Vienna, Austria

**Keywords:** Ovarian stimulation, Polycystic ovary syndrome, Endometrium thickness at midcycle, Clomiphene, AMH, LH/FSH ratio, PCOS, Infertility

## Abstract

**Purpose:**

Recent studies reported that in polycystic ovary syndrome (PCOS) patients, other stimulation agents are superior to the popular first-line regimen, clomiphene citrate (CC) for ovarian stimulation. Nonetheless, CC is still widely used since it is not clear which patients will not respond to it. Furthermore, the prognostic value of endometrium thickness at midcycle is controversial. We aimed to find factors predicting the response to CC and the prognostic value of endometrial thickness at midcycle.

**Methods:**

We collected data retrospectively from 89 anovulatory PCOS patients who had the first stimulation with 50 mg CC. We analyzed the basal levels of AMH, testosterone, LH, LH:FSH ratio and the endometrial thickness at midcycle by univariate, followed by multivariate regression. The outcome measures were pregnancy, follicle maturation and endometrial thickness at midcycle.

**Results:**

Stimulation with 50 mg CC resulted in follicle maturation in 50.6% of the women and in 27.0% pregnancies. In the univariate analysis, greater endometrial thickness, lower LH and AMH levels and a lower LH:FSH ratio were associated with pregnancy (*p* < 0.05). In the multivariate analysis, only endometrial thickness remained predictive (*p* = 0.045). The endometrial thickness cutoff level of ≥ 8 mm showed a sensitivity of 87.5% (96% CI 67.6–97.3) and a specificity of 66.7% (95% CI 43.0–85.4) for prediction of pregnancy. In the multivariate analysis AMH levels 5.4 (3.4; 7.0) (ng/mL) predicted pregnancy (*β* = − 0.194 ± 0.092; *p* = 0.034)

**Conclusion:**

We suggest to refrain from CC as first-line regimen in patients with AMH > 7 ng/ml. Under CC treatment, the cutoff value of ≥ 8 mm endometrium thickness at midcycle is associated with a better outcome.

## Introduction

Polycystic ovary syndrome (PCOS), a highly frequent endocrine disorder which affects at least 6–10% of women in reproductive age [1, 2] is the main cause of infertility due to anovulation [2]. Generally, clomiphene citrate (CC) is the favored first-line agent for ovulation induction in PCOS-related infertility [2] for the majority of gynecologists [3]. However, evidence has suggested that stimulation with letrozole would be superior in terms of pregnancy and life birth rates, as shown by recent meta-analyses of randomized controlled trials [4, 5] as well as recommendations from the international evidence-based six continent international advisory guideline [6]. Clomiphene citrate is a selective estrogen modulator (SERM) known to negatively impact endometrial development [7, 8]. A recent study found that treatment with CC was associated with dysregulation of the Wnt signaling pathway not only in PCOS but also in healthy women [9]. Oftentimes, CC stimulation results in a thinner endometrium at ovulation. This effect of CC on endometrium has been held accountable for the worse fertility outcomes compared with other stimulation agents including letrozole [10].

Nonetheless, CC still remains the most popular treatment regimen among gynecologists. This could be partly due to the fact that the use of letrozole for ovarian stimulation is off-label in many countries. Moreover, some women show a better response to letrozole, some to CC. In terms of personalized medicine, it would be helpful to know in advance which factors predict a success and to tailor the regimen to the individual patient accordingly. Given that at times CC results in low endometrium thickness (EMT) presumably lowering pregnancy rate, one first step would be to test the hypothesis of whether EMT at midcycle is associated with a better outcome and to find out the optimal EMT value that predicts pregnancy for infertile PCOS women stimulated with CC. If a certain endometrial thickness cutoff is indeed associated with better outcomes, the next step would be to find factors that predict this favorable development.

PCOS is associated with substantially increased serum levels of AMH [11]. Notably, receptors for Anti-Mullerian hormone (AMH) have been identified in adult human endometrium [12]. Through its AMH-II receptor, this substance seems capable of negatively regulating cellular viability both in cells of normal endometrium and endometriosis [12–14]. Therefore, a link between insufficient endometrial development during CC stimulation and AMH serum levels can be assumed.

It seems plausible that in addition to the already altered expression of endometrial receptivity markers in PCOS patients [15] and the detrimental effects of CC on the endometrium [9], the higher AMH levels might further deteriorate the local milieu for the successful implantation and early development of the pregnancy. Despite this, most studies examining the efficacy of the CC treatment have the ovulation rate as the primary outcome, thus ignoring the essential role of endometrium for a successful outcome in PCOS patients [16]. Published high-level evidence studies aimed at finding models of prognostic factors for PCOS-related infertility did not consider basal AMH levels along other parameters [17–19]. Furthermore, there is some controversy regarding the importance of EMT at midcycle and its prognostic value in PCOS women. Most studies that have examined the importance of EMT for pregnancy outcome after IVF/ICSI are also controversial [20, 21]. The EMT at midcycle seems to not matter in women undergoing intrauterine insemination after CC stimulation [22], although the relevance of this particular study to the PCOS patients is not clear since the majority of women in this study did not have PCOS. Despite the evidence that CC stimulation in PCOS patients is associated with lower mid-cycle EMT and pregnancy rate [10] compared to Letrozole, it is still not clear whether within the PCOS patient collective stimulated with CC, there is difference in pregnancy outcome based on mid-cycle EMT.

Thus, in this retrospective analysis on infertile, anovulatory PCOS women, we focused on two major issues: (i) evaluation of an optimal cutoff for endometrial thickness at the time of ovulation induction after CC stimulation to predict pregnancy; and (ii) evaluation of predictive factors—including AMH among other markers for PCOS-severity—for endometrial thickness, for a better outcome and to shed some light on pathophysiologic mechanisms responsible for endometrial development in PCOS women during CC stimulation.

## Methods

### Study population

In this retrospective analysis, we included 89 consecutive anovulatory, infertile women with PCOS who underwent their first cycles of stimulation with 50 mg CC for 5 days at our department from January 2016 until December 2017. PCOS was diagnosed according to the revised European Society of Human Reproduction and Embryology (ESHRE) and the American Society for Reproductive Medicine (ASRM) criteria of 2003 [23, 24]. The diagnosis was made in the event that two out of the three criteria were fulfilled: oligo/anovulation, clinical and/or biochemical signs of hyperandrogenism and polycystic ovaries. All women revealed ≥ 12 follicles of 2–9 mm diameter on at least one ovary on transvaginal ultrasound, as well as 17-hydroxy progesterone levels < 2 ng/mL, and, thus, non-classical adrenogenital syndrome could be excluded [25].

None of the women had ever undergone any other kind of ovarian stimulation or laparoscopic ovarian drilling.

The main outcome measures were follicle maturation and clinical pregnancy after CC stimulation as well as endometrial thickness as measured by transvaginal ultrasound on the day of ß-HCG administration for ovulation induction. In addition to this, the following parameters were also collected and included in the analysis as independent variables: patients’ age, body mass index (BMI), type of sterility (primary/secondary), parity and PCOS-specific co-medications including treatment with metformin or myo-inositol, luteinizing hormone (LH), follicle-stimulating hormone (FSH), the LH:FSH ratio, testosterone, and AMH. All blood samples were taken during the early follicular phase visit (cycle days 2–5) before the start of CC stimulation cycle. All serum parameters were determined using commercially available assays.

### Ultrasound measurement and treatment protocol

The endometrium was measured with an Ultrasound Machine Model Aloka Prosound 6 (Hitachi Medical Systems). The measurements were performed by one of three reproductive endocrinology specialists in the median sagittal longitudinal plane of the uterus at the maximum distance between the endometrial–myometrial interface of the anterior to the posterior wall of the uterus. The endometrium thickness was first assessed with a baseline TVUS on day 3, following the onset of spontaneous or progestogen-induced menstrual cycle. Since studies have shown no difference in outcome between starting treatment on day 2, 3, 4 or 5 [26], our patients were prescribed 50 mg CC as from day 5–9 of the cycle. The recommended starting dose is 50 mg/day, as almost half of the pregnancies are achieved with this dose [27] and is the first-line treatment recommended by the Endocrine Society [28]. Beginning with the 10th day of the menstrual cycle up until the 14th day of the menstrual cycle, all patients underwent daily monitoring of follicular growth using vaginal sonography. After the patients emptied their bladders, the endometrial thickness and pattern were assessed by transvaginal 8 MHz ultrasonography. As soon as the leading follicle reached a mean diameter of 19 mm and the growth of endometrium showed a trilaminar fashion, the final oocyte maturation was induced by 10,000 IU of HCG intramuscular application of human chorionic gonadotrophin. In the event that two follicles developed, patients were informed about the increased risk of multiple pregnancy. Since none of the patients chose to interrupt the stimulation cycle, ovulation was induced. Within the study period, none of the patients developed three or more follicles. Timed intercourse was advised, all women tried to conceive naturally and there were no cycles of intrauterine insemination. Beginning on the day after ovulation induction, all women received dydrogesterone 20 mg, administered for 10 days.

### Statistical analysis

Variables are described by numbers (frequencies) and median and interquartile ranges (IQR). Binary logistics regression models were used to test for the explanatory power of parameters on categorical outcomes. In a first step, all parameters were tested univariately, all significant predictors were then included in multivariate models. Possible influencing factors on numerical outcomes, namely endometrial thickness, were tested using generalized linear models. In a generalized linear model, the impact of several parameters on endometrial thickness was evaluated. For these analyses, regression coefficients *β* and their standard deviations (SD) as well as likelihood ratio tests are provided. The optimized cutoff value for prediction of pregnancy by use of endometrial thickness was defined as the one with the highest sum of sensitivity and specificity. Contingency tables were used to calculate sensitivity, specificity, positive and negative predictive values. A Fisher’s exact test was performed to test for statistical significance in this model. Statistical analysis was performed with SPSS 24.0 for Windows (SPSS Inc, 1989–2018). A post-hoc power analysis for logistic regression models was performed using the open access software R [29]. Differences were considered statistically significant if *p* < 0.05.

## Results

General patient and PCOS characteristics are shown in Table 1. The first stimulation cycle with 50 mg CC resulted in follicle maturation in 45 women (50.6%) and in a pregnancy in 24 (27.0%). In the analysis about the impact of endometrial thickness on the chance of pregnancy, we focused on women with follicle maturation only. In univariate followed by multivariate analyses, putative predictive parameters for the achievement of clinical pregnancy were tested (Table 2). In the univariate analyses higher endometrial thickness, lower LH and AMH levels along with lower LH:FSH ratio were associated with higher pregnancy rates (*p* < 0.05). When all these parameters were tested together in a multivariate manner, only endometrial thickness remained significantly predictive (*p* = 0.045). For this analysis, a post-hoc power analysis was performed. Assuming an alpha error of 5%, an odds ratio of 1.76 for EMT (which accords to the observed *β*) and a mean EMT of 9.02 ± 2.3, a power of 96.9% was calculated.

**Table 1:** Basic patient characteristics and PCOS-specific serum parameters

Age (years)*	29.1 (26.4; 33.3)
BMI (kg/m^2^)*	25.8 (23.3; 29.7)
Primary sterility^#^	23 (25.8)
Concomitant use of metformin^#^	24 (27.0)
Concomitant use of myo-inositol^#^	12 (13.5)
LH (mU/mL)*	10.6 (8.0; 14.2)
FSH (mU/mL)*	5.5 (4.8; 6.7)
LH:FSH ratio*	2.0 (1.5; 2.5)
Testosterone (ng/mL)*	0.42 (0.28; 0.52)
AMH (ng/mL)*	7.8 (5.0; 12.7)

Data are provided as *median (IQR) or ^#^number (frequency)

**Table 2:** Predictive parameters for pregnancy in women with follicle maturation (*n* = 45)

Parameter	Pregnancy (*n* = 27)	No pregnancy (*n* = 18)	Univariate analysis		Multivariate analysis	
			*β* (SD)	*p*	*β* (SD)	*P*
Age (years)*	30.9 (26.5; 34.2)	29.3 (27.0; 31.0)	0.081 (0.068)	0.233	–	–
BMI (kg/m^2^)*	25.6 (21.9; 30.3)	25.7 (20.4; 29.0)	– 0.002 (0.063)	0.970	–	–
Primary sterility^#^	17 (70.8)	16 (76.2)	0.276 (0.681)	0.686	–	–
Concomitant use of metformin^#^	6 (22.2)	5 (27.8)	– 1.460 (0.763)	0.056	–	–
Concomitant use of myo-inositol^#^	6 (22.2)	1 (5.6)	– 0.499 (0.831)	0.548	–	–
Endometrial thickness (mm)*	10.1 (9.0; 11.0)	7.8 (7.0; 8.5)	0.566 (0.186)	0.002	0.412 (0.175)	0.045
LH (mU/mL)*	8.3 (6.1; 9.9)	12.7 (8.3; 15.0)	– 0.204 (0.085)	0.017	– 0.054 (0.157)	0.730
FSH (mU/mL)*	5.8 (4.8; 6.7)	5.8 (4.7; 7.2)	– 0.011 (0.197)	0.995	–	–
LH:FSH ratio*	1.5 (1.0; 1.7)	2.3 (1.6; 2.9)	– 1.005 (0.441)	0.023	– 0.540 (0.132)	0.504
Testosterone (ng/mL)*	0.35 (0.25; 0.47)	0.42 (0.32; 0.50)	– 3.547 (2.297)	0.122	–	–
AMH (ng/mL)*	5.4 (3.4; 7.0)	10.0 (6.8; 13.1)	– 0.365 (0.126)	0.004	– 0.247 (2.441)	0.061
Growth of two follicels^#^	2 (7.4)	1 (5.6)	0.644 (1.264)	0.610	–	–

Data are provided as *median (IQR) or ^#^number (frequency)

Results of the univariate followed by multivariate analyses

**Fig. 1:**
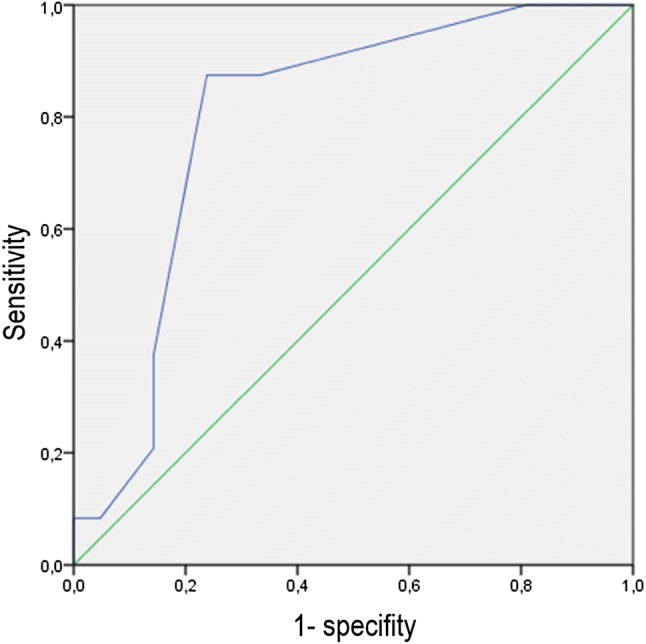
Receiver operator characteristic (ROC) curve for the prediction of pregnancy by means of endometrial thickness

Thus, we calculated the optimal cutoff level of endometrial thickness for the prediction of pregnancy. The corresponding receiver operator characteristic (ROC) curve is shown in Fig. 1. The optimized cutoff value, i.e., the one with the highest sum of sensitivity and specificity, was ≥ 8 mm. The use of this cutoff level was associated with a sensitivity of 87.5% (96% CI 67.6; 97.3), a specificity of 66.7% (95% CI 43.0; 85.4), a positive predictive value of 75.0% (55.1; 89.3) and a negative predictive value of 82.4% (56.6; 96.2; *p* < 0.001 in Fisher’s exact test).

The optimized cutoff value, i.e., the one with the highest sum of sensitivity and specificity, was ≥ 8 mm. The use of this cutoff level was associated with a sensitivity of 87.5% (96% CI 67.6; 97.3), a specificity of 66.7% (95% CI 43.0; 85.4).

In a last step, a generalized linear model was used to test search for parameters that had influenced endometrial thickness (Table 3). In this analysis, only lower AMH serum levels were significantly associated with higher endometrial thickness at the time of ovulation induction (*p* < 0.001).

**Table 3:** Predictive parameters for endometrial thickness

Parameter	*β* (SD)	*P*
Age (years)	– 0.111 (0.0699)	0.111
BMI (kg/m^2^)	0.029 (0.0658)	0.661
LH (mU/mL)	0.027 (0.2533)	0.916
FSH (mU/mL)	– 0.673 (0.5441)	0.216
LH:FSH ratio	– 0.450 (1.3392)	0.737
Testosterone (ng/mL)	– 3.041 (2.2225)	0.171
AMH (ng/mL)	– 0.241 (0.0737)	0.001
Constant	19.120 (4.2810)	< 0.001

Results of a generalized linear model

AMH and other parameters have been proposed to be good predictive parameters for follicle maturation during CC stimulation in PCOS women [16, 30, 31] and endometrial thickness, which was associated with certain serum AMH levels, seemed predictive for the pregnancy rate after CC stimulation in a similar patient population. Due to this, we evaluated predictive parameters for overall outcome in terms of pregnancy rates in an additional analysis (Supplementary Table 1). Endometrial thickness was not included in this analysis because it was only available for women who had achieved follicle maturation. In the univariate analyses, higher LH, testosterone, and AMH levels as well as a higher LH:FSH ratio were associated with lower pregnancy rates. In the multivariate analysis, only AMH remained significantly predictive of CC stimulation success (*β* = − 0.194 ± 0.092; *p* = 0.034).

## Discussion

We aimed to contribute data with the final goal of finding a first-line treatment algorithm for ovarian stimulation in PCOS. We consider the baseline characteristics robust despite the small sample size. Taking the initial patient population into account which additionally included all women who did not develop a follicle after CC stimulation, the pregnancy rate achieved was about 30% which is considerably high when compared with the previous literature [10, 32, 33]. We presume that this result might be due to the design of our study. To our knowledge, there are no studies reporting the success rate of clomiphene at the first cycle only in a cohort of anovulatory PCOS patients, naïve to ovulation stimulation. Another possible explanation for the unusually high success rate is the small sample size.

In 20% of all women stimulated with CC 50 mg (18/89), pregnancy could not be achieved despite follicular growth. This subgroup of women, compared to the group where treatment resulted in both follicular growth and subsequent clinical pregnancy (27/89), had significantly lower EMT at midcycle, higher basal AMH levels, lower LH levels and a lower LH:FSH ratio. However, after multivariate analysis, only EMT remained predictive for pregnancy in women with follicular growth (Table 2). Thus, EMT evidently played a major role in our patient population and an optimized cutoff level of ≥ 8 mm could be computed. As stated previously, CC stimulation might result in thinner endometrium at the time of ovulation compared to other stimulation regimens including letrozole [10]. Our data are in line with other studies that have pointed out an association of EMT with clinical pregnancy rate and abortion rate, at least in patients after in vitro fertilization (IVF). Richter et al. [34] for instance, found that the pregnancy rate was higher in cycles with increasing EMT (11.9 vs 11.3 mm, respectively). In a meta-analysis of 22 studies examining the impact of EMT as a tool to decide on cycle cancellation in IVF treatment, Kasius et al. demonstrated that an EMT ≤ 7 mm was associated with significantly poorer outcome, although observed in only a very limited percentage of cycles, namely 2.4% [35].

When focusing on CC stimulation, one study has led to an interesting finding: in women with unexplained infertility after three cycles of failed stimulation, an EMT of ≤ 7 mm (but > 5.5 mm) was associated with the best pregnancy rates, while EMT ≥ 11.6 mm with the worst [30]. However, as reviewed in a recent meta-analysis of 23 studies that had compared different ovarian stimulation agents such as CC, gonadotropin or aromatase inhibitors prior to IUI in women with unexplained subfertility, there was no association between EMT and pregnancy rates [36]. Thus, we postulate that the optimal cutoff for EMT of ≥ 8 mm midcycle applies primarily to the setting of anovulatory PCOS patients and that this is not the “magical cut-off” that should be implemented in other settings of infertility and CC stimulation.

We find it crucial to put an emphasis on the association of high serum AMH levels and lower EMT at the time of ovulation induction (Table 3). As previously mentioned, AMH receptors have been identified in adult human endometrium [12] (10). AMH is thought to exert negative effects on cellular viability [12–14] by binding these receptors. The results of our study are in line with these observations. Wang et al. have proposed that AMH that binds to its endometrial receptors might derive from autocrine or paracrine sources [12]. However, hypothetically speaking, this might not be true for women with supraphysiologic AMH serum levels as is the case for PCOS patients [11].

Not having taken into consideration the mid-cycle estradiol levels might be a study limitation, since they might have influenced the EMT. However, CC treatment is not only associated with an increase in estradiol levels but also with a decrease in estrogen receptor expression in the endometrium [37] which along with the dysregulation of Wnt signalling pathway, might contribute to a suboptimal development of the endometrium. In addition, in the decision-making process between CC and letrozole stimulation, only parameters available at the beginning of the cycle are of interest. Thus, the mid-cycle estradiol levels would have not been helpful in the initial baseline decision of whether or not to stimulate with CC or other agents.

Notably, BMI was not a prognostic factor for pregnancy in our data set, neither in those women who achieved follicle maturation (Table 2), nor in the whole data set (Suppl. Table 1). The average BMI for all women in our analysis was 25.8 kg/m^2^ which is representative of the Austrian population of this age-group [38]. Despite this, some studies reported that the high BMI played a role in the CC resistance with regard to ovulation rate [16]*.* Due to the fact that the majority of our PCOS patients had a normal BMI, it is possible that our study was underpowered to detect the BMI’s significant impact.

It was not the primary aim of our study to predict overall CC outcome. However, we presented a predictive model in the Supplementary Table 1. Since in women with follicle maturation lower AMH levels were associated with higher EMT and due to the fact that the latter was predictive for achievement of pregnancy, it is not surprising that AMH was the most important predictive parameter in our multivariate model of all women who underwent CC stimulation (Suppl. Table 1). The effect of AMH outweighed those of LH, the LH:FSH ratio, and testosterone in our study. Our findings confirm once more that high AMH levels were predictive of a negative outcome after CC stimulation in PCOS women [16, 30, 31, 34]. This seems plausible considering AMH’s main role in disruption of folliculogenesis in PCOS [39].

In addition to considerations about possible study limitations, one weakness of the study was the small sample size. Due to this the study might have been unable to detect subtle prognostic factors. However, for the significant parameter EMT a post-hoc power of 96.9% was calculated which likely excludes type I and II errors. Thus, the robustness of the positive finding that the AMH levels at baseline predict the optimal EMT at mid-cycle and pregnancy rate should not have been weakened by the small sample size.

## Conclusions

Higher serum AMH levels 9.1 (6.6; 14.6) versus 5.4 (3.4; 7.0) ng/mL were predictive for lower EMTafter CC stimulation in anovulatory PCOS women and a lower chance to achieve pregnancy in these patients. In patients who achieved follicle maturation, an EMT < 8 mm was significantly associated with a lower probability of pregnancy. Thus, one might recommend refraining from using CC as a first-line regimen in PCOS anovulatory patients with AMH levels in the higher range. Prospective studies are warranted to confirm our findings in larger patient populations.

## Abbreviations

AMH: Anti Mullerian hormone
BMI: Body Mass Index
CC: Clomiphene citrate
EMT: Endometrial thickness
FSH: Follicular stimulating hormone
IQR: Interquartile ranges
IVF: In vitro fertilization
LH: Luteinizing hormone
PCOS: Polycystic ovary syndrome
SD: Standard deviation
TVUS: Transvaginal ultrasound
